# Exploring alterations in sensory pathways in migraine

**DOI:** 10.1186/s10194-021-01371-y

**Published:** 2022-01-12

**Authors:** Noemi Meylakh, Luke A. Henderson

**Affiliations:** School of Medical Sciences (Neuroscience), Brain and Mind Centre, University of Sydney, Camperdown, NSW 2050 Australia

**Keywords:** Migraine, Infra-slow oscillations, Functional connectivity, Primary visual cortex, Secondary visual cortex, Sensory network

## Abstract

**Background:**

Migraine is a neurological disorder characterized by intense, debilitating headaches, often coupled with nausea, vomiting and sensitivity to light and sound. Whilst changes in sensory processes during a migraine attack have been well-described, there is growing evidence that even between migraine attacks, sensory abilities are disrupted in migraine. Brain imaging studies have investigated altered coupling between areas of the descending pain modulatory pathway but coupling between somatosensory processing regions between migraine attacks has not been properly studied. The aim of this study was to determine if ongoing functional connectivity between visual, auditory, olfactory, gustatory and somatosensory cortices are altered during the interictal phase of migraine.

**Methods:**

To explore the neural mechanisms underpinning interictal changes in sensory processing, we used functional magnetic resonance imaging to compare resting brain activity patterns and connectivity in migraineurs between migraine attacks (*n* = 32) and in healthy controls (*n* = 71). Significant differences between groups were determined using two-sample random effects procedures (*p* < 0.05, corrected for multiple comparisons, minimum cluster size 10 contiguous voxels, age and gender included as nuisance variables).

**Results:**

In the migraine group, increases in infra-slow oscillatory activity were detected in the right primary visual cortex (V1), secondary visual cortex (V2) and third visual complex (V3), and left V3. In addition, resting connectivity analysis revealed that migraineurs displayed significantly enhanced connectivity between V1 and V2 with other sensory cortices including the auditory, gustatory, motor and somatosensory cortices.

**Conclusions:**

These data provide evidence for a dysfunctional sensory network in pain-free migraine patients which may be underlying altered sensory processing between migraine attacks.

## Background

Migraine is an incapacitating neurological disorder consisting of recurring headaches accompanied by sensory, autonomic, cognitive, and affective symptoms [1]. One third of migraineurs experience focal transient symptoms of visual aura preceding the onset of a migraine headache [2], and most migraineurs also experience photophobia, whereby headaches are exacerbated by light or whereby the individual experiences an abnormal sensitivity to light [3, 4]. In addition, migraine attacks are associated with abnormalities in multiple sensory modalities, including audition, olfaction and somatosensation [5], manifested through symptoms such as phonophobia, osmophobia and cutaneous allodynia respectively [6].

Whilst changes in sensory processing during a migraine is well-described, it is increasingly evident that altered sensory processing also occurs between migraine attacks [5, 7]. Evidence suggests that during the interictal phase, most migraineurs display diminished thresholds for light, sound and odors as well as reduced visual and auditory discomfort thresholds [8–10]. It is known that in many migraineurs, noise, light and smell can trigger a migraine attack [11, 12]. Compared with controls, migraineurs also display altered somatosensory processing characterized by reduced thermal and mechanical pain thresholds between migraine attacks [13]. Altered integration of unimodal sensory processing is also a characteristic of migraine with exposure to one sensory modality altering the sensitivity of another. For example, olfactory and visual hypersensitivities and auditory and somatosensory hypersensitivities are often coupled [12, 14]. These reports strongly suggest that even during the interictal phase, brain circuits that process visual, auditory, olfactory and somatosensory information displayed altered sensitivities and also increased coupling compared with non-migraine controls.

Whilst brain imaging studies have revealed altered coupling between areas of the descending pain modulatory pathway (including the midbrain periaqueductal gray (PAG) and spinal trigeminal nucleus (SpV)), as well as between somatosensory processing regions in interictal migraine [15, 16], coupling between individual sensory cortical regions has not been investigated. In order to understand the underlying mechanism of migraine, the nature of these atypical sensory processing networks between migraine attacks needs to be further investigated. We have previously reported changes in resting activity levels and patterns in the visual cortex of migraineurs [17], and we aim to determine if ongoing functional connectivity patterns between visual, auditory, olfactory, gustatory and somatosensory cortices are altered during the interictal phase. We hypothesize that the visual cortex will display altered resting on-going activity patterns, measured as increased infra-slow oscillations, and that the on-going activity within these visual areas will exhibit increased functional coupling with other cortical sensory processing regions.

## Methods

### Subjects

Thirty-two subjects with episodic migraine (24 females; mean ± SEM age 28.77 ± 1.5 years) and 71 pain-free controls (63 females; mean ± SEM age 30.19 ± 1.3 years) were recruited for the study from the general population using a flyer. In a previous series of chronic pain investigations, we assessed resting state connectivity and infra-slow oscillations and found that significantly different regions had an effect size (Cohen’s D) of at least 0.83. We expected similar levels of change in this study and power analysis suggests that we needed 30 subjects in each group to detect even the smallest effect size. Given this we recruited more than 30 migraineurs and 30 controls to take part in this investigation. Migraine subjects were diagnosed according to the International Headache Society (IHS) classification of The International Classification of Headache Disorders 3rd edition (ICHD-3) beta criteria and their characteristics are shown in Table 1. Eight of the 32 migraine subjects reported an accompaniment of visual aura, and 29 reported photophobia/light sensitivity during their migraine attacks. All migraineurs were scanned during a pain-free interictal period; i.e., not within 72 h following or 24 h preceding a migraine attack.

**Table 1:** Migraine subject characteristics

Subject	Age	Sex	Years suffering	Pain side	Aura	Migraine attack/ month	Intensity (0–5)	Migraine medications
1	31	F	25	R	Y	5–8	3–4	paracetamol
2	24	F	20	B	N	4	4	ibuprofen, paracetamol
3	26	F	12	R	N	2	3–4	ibuprofen
4	27	F	12	R	Y	1	4	ibuprofen
5	23	F	4	R	N	4	4	triptan
6	25	F	12	L	N	5	3	aspirin, rizatriptan
7	21	F	1.5	L	N	4	3	ibuprofen, paracetamol
8	25	F	1	L	N	3	5	paracetamol
9	29	F	13	R	N	1	2.5	ibuprofen
10	26	F	5	R	N	1	2	aspirin, ibuprofen, codeine
11	23	F	6	R	N	1	3–4	ibuprofen
12	23	F	10	B	N	0.5–1	4	ibuprofen, codeine
13	46	F	15–20	B	N	1	3	sumatriptan
14	41	F	40	B	N	2	4	sumatriptan
15	26	M	15	B	N	8	3	TCE, paracetamol, codeine
16	23	M	3–4	B	N	0.5–1	3.5	paracetamol, codeine
17	23	M	4–5	B	N	0.5–1	4	paracetamol
18	55	F	40	R	N	0.5–1	3–4	sumatriptan
19	26	M	20	R	N	0.5–1	4	metamizole
20	49	F	30	B	N	0.5–1	5	rizatriptan, paracetamol
21	34	F	15	L	Y	2	3	ibuprofen, paracetamol
22	26	F	5	B	Y	1	3	paracetamol
23	25	F	7–8	L	N	5–8	3	rizatriptan, benzoate
24	27	M	4	B	N	0.5–1	4	ibuprofen
25	28	F	25	R	Y	0.25	5	ibuprofen
26	25	M	6	L	N	0.5–1	4	paracetamol, codeine forte
27	24	F	13	R	Y	3–4	5	TCL, paracetamol, codeine
28	19	F	4–5	B	N	3–4	3	lexapro
29	25	M	12	L	N	2	4	paracetamol
30	26	F	9	R	Y	1	3	ibuprofen
31	27	F	10–12	R	N	0.3	3	–
32	44	M	0.25	B	Y	1	5	ibuprofen, paracetamol

*M* male, *F* female, *B* bilateral, *L* left, *R* right, *I* interictal, *OCP* oral contraceptive pill, *SSRI* selective serotonin reuptake inhibitor

Control subjects were excluded if they presented with any pain condition, neurological disorder, current use of analgesics or a family history of migraine. Migraineurs were excluded if they presented with any other pain or neurological condition other than migraine. No subjects.

had an incidental neurological finding. Each migraineur rated the intensity of their migraine pain on a six-point visual analogue scale (0 = no pain; 5 = most intense pain imaginable) and drew the location of their migraine attacks on a facial distribution map. Self-reported information pertaining to the location, quality and intensity of migraines, including medications are listed in Table 1. According to the Declaration of Helsinki, informed written consent was obtained for all procedures. The local Institutional Human Research Ethics committee approved the study. Data from 26 of the 32 migraine subject patients and all control subjects have been used in our previous studies [17, 18].

### MRI acquisition

Subjects lay supine on the bed of a 3 T MRI scanner (Philips, Achieva) with their head restrained in a tight-fitting head coil. With each subject relaxed and at rest, a high-resolution 3D T1-weighted anatomical image set, covering the entire brain, was collected (turbo field echo; field of view = 250 × 250 mm, matrix size = 288 × 288, slice thickness = 0.87 mm, repetition time = 5600 ms; echo time = 2.5 ms, flip angle = 8°). Following this, subjects were instructed to keep their minds as clear and relaxed as possible as a series of 180 gradient echo echo-planar functional MRI (fMRI) image volumes using blood oxygen level dependent (BOLD) contrast were collected. Each image volume contained 38 axial slices covering the entire brain (field of view = 240 × 240 mm, matrix size = 80 × 78, slice thickness = 4 mm, repetition time = 2000 ms; echo time = 30 ms, flip angle = 90°).

### MRI data processing and statistical analysis

#### Image preprocessing

Using Statistical Parametric Mapping version 12 (SPM12) [19] and custom Matlab software, all fMRI images underwent slice timing correction and motion correction. We detected no significant movement greater than 0.5 mm in any direction. Movement parameters were modelled and removed from the fMRI signal by LMRP detrending. The Bayesian method for physiological noise correction, the Dynamic Retrospective Filtering (DRIFTER) algorithm [20], was used to reduce potential effects of physiological noise. Using the DRIFTER algorithm, a cardiac frequency band of 60–120 beats per minute (+ 1 harmonic) and a respiratory frequency band of 8–25 breaths per minute (+ 1 harmonic) were removed. Following this, the fMRI images were then LMGS detrended to remove high-frequency global signal fluctuations [21] and each subject’s fMRI image set was co-registered to their own T1-weighted anatomical image. The T1 image was then spatially normalized to the Montreal Neurological Institute (MNI) template and the parameters applied to the fMRI image set. The anatomical locations of significant clusters were confirmed using the Atlas of the Human Brain by Mai, Paxinos, and Voss [22].

#### Infra-slow oscillation power

Using the DPARSFA toolbox [23], raw amplitude of low-frequency fluctuation analysis (ALFF) power was calculated between 0.03 and 0.06 Hz for each voxel of each image set in the control and migraine subjects. This frequency band was chosen since we have previously shown that power in this specific band is altered in migraine and a number of other pain conditions [17, 24, 25]. The resulting brain maps were then smoothed using a 3 mm full-width half maximum (FWHM) Gaussian filter and analysis restricted to the occipital lobe. Significant voxel-by-voxel differences in 0.03–0.06 Hz power between controls and migraineurs were determined using a two-sample random effects procedure (p < 0.05, corrected for multiple comparisons, minimum cluster size 10 contiguous voxels, age and gender included as nuisance variables). Significant differences were then overlaid onto a mean T1-weighted anatomical image, derived from all 103 subjects, for display purposes. The locations of clusters were defined using the “Probabilistic Maps of Visual Topography in Human Cortex” by Wang and colleagues [26]. Finally, for each significant cluster, ALFF values were extracted from each subject and individual as well as the mean ± SEM values plotted to indicate direction and strength of ALFF power.

#### Functional connectivity

Resting functional connectivity was conducted using four clusters derived from the ALFF analysis, right primary visual (V1), right secondary visual (V2), right third visual (V3) and left V3 cortices. We firstly smoothed the fMRI images sets using a 6 mm FWHM Gaussian filter. Then for each of the three seeds, signal intensity changes were extracted from each voxel in each subject and averaged. For each subject the signal intensity timetrend was then entered into a first level analysis and individual subject brain maps created with each voxel value representing the correlation strength and direction with respect to the seed signal intensity changes. To identify significant differences in connectivity strengths between controls and migraineurs, the connectivity maps were placed into a second-level, random-effects analysis with (*p* < 0.05, corrected for multiple comparisons, minimum cluster size 10 contiguous voxels, age and gender as nuisance variables). Significant connectivity strength differences for each of the seeds were then overlaid onto a mean T1-weighted anatomical image. To determine the overlap of connectivity differences between controls and migraineurs, the brain maps of two analyses that resulted in significant differences were binarized and added together. Finally, for clusters in the resulting overlap brain map, beta values indicating the correlation strength effect size were extracted from each subject for each of the seed maps and individual and mean ± SEM values plotted to indicate direction and strength of relationships.

The data presented in this study are available on request from the corresponding author. The data are not publicly available due to ethical reasons.

## Results

### Infra-slow oscillations

**Fig. 1:**
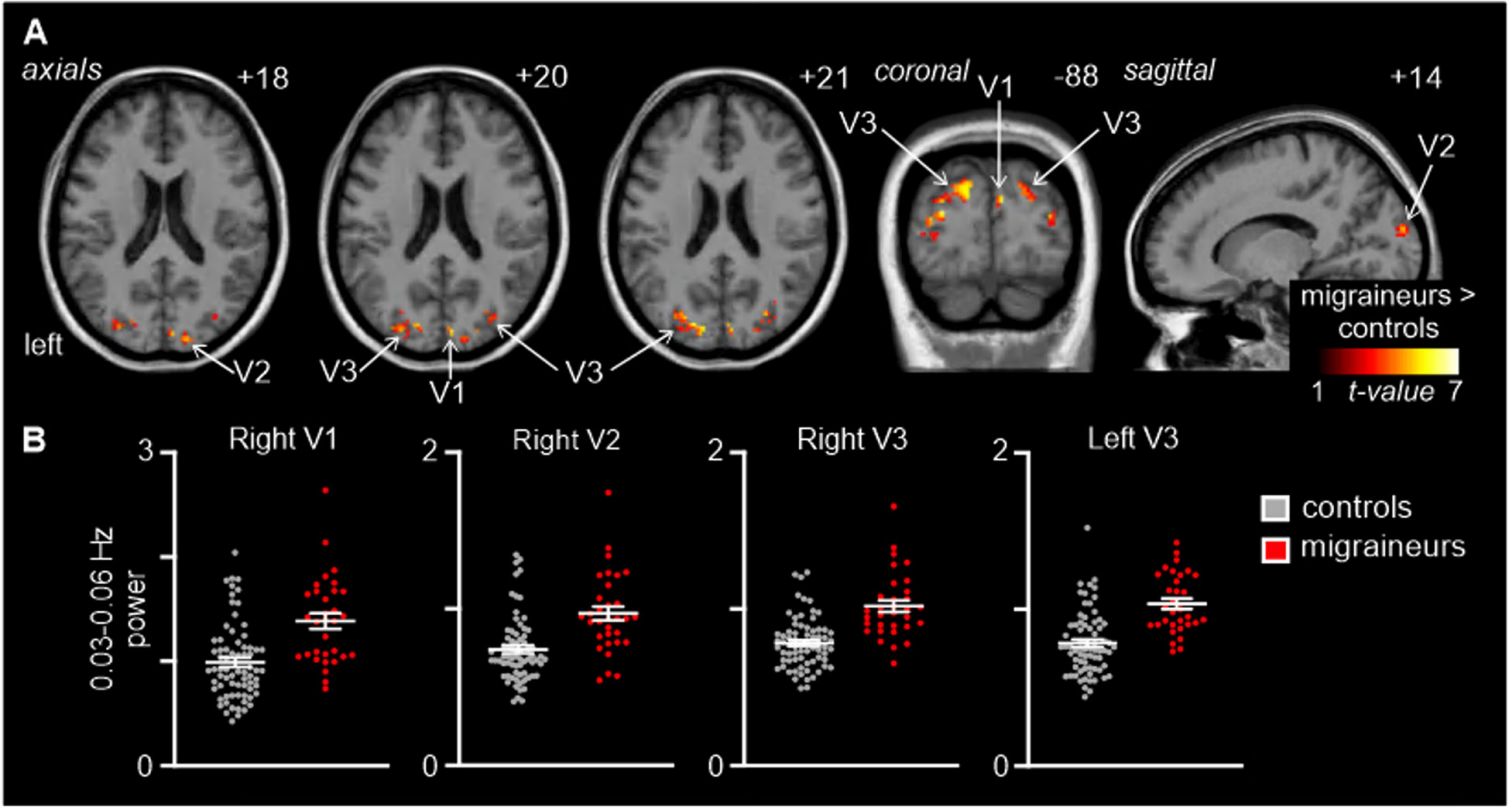
**A**. Significantly higher resting infra-slow (0.03–0.06 Hz) power in migraineurs (*n* = 32) compared with controls (*n* = 71) in the occipital lobe (random effects, *p* < 0.05, corrected). Significant clusters are overlaid onto a mean T1-weighted anatomical image and the location of each axial, coronal and sagittal slice in Montreal Neurological Institute space indicated at the top right of each slice. **B**. Plots of individual subject and mean (± standard error of mean) power in four significant clusters. *V1: primary visual cortex; V2: secondary visual cortex; V3: third visual complex*

Comparison of resting 0.03–0.06 Hz power revealed a number of clusters in the occipital lobe in which power was signficantly different between controls and migraineurs (Fig. 1; Table 2). Significantly higher infra-slow oscillatory power occurred in migraineurs in the right V1 (mean ± SEM 0.03–0.06 Hz power: controls: 0.99 ± 0.04, migraineurs: 1.38 ± 0.07), right V2 (controls 0.74 ± 0.02, migraineurs 0.97 ± 0.04) right V3 (controls 0.78 ± 0.02, migraineurs 1.01 ± 0.04), and left V3 (controls 0.79 ± 0.02, migraineurs 1.04 ± 0.03). In no region was ALFF power significantly lower in migraineurs compared with controls. There were no significant differences in age (t-test; *p* < 0.05) or gender composition (chi-squared test; *p* < 0.05) between the two groups.

**Table 2:** Information on brain regions exhibiting significant differences

Brain region	MNI coordinates			Cluster size	t score
	x	y	z		
*Infra-slow oscillation power*					
*Migraineurs > Controls*					
Right primary visual cortex	6	−88	20	12	5.78
Right secondary visual cortex	20	− 92	16	34	5.79
Right third visual complex	24	− 82	26	136	6.54
Left third visual complex	−24	−82	28	198	6.61
*V1 resting functional connectivity*					
*Migraineurs > Controls*					
Primary auditory cortex	60	−6	−4	95	4.51
Left secondary auditory cortex	− 52	− 44	8	293	5.45
Right secondary auditory cortex	62	−32	6	171	4.65
Primary somatosensory cortex	44	−20	36	220	5.23
Left secondary somatosensory cortex	−38	−4	10	158	4.56
Right secondary somatosensory cortex	58	−10	20	78	4.41
Left primary motor cortex	− 42	4	34	14	3.82
Right primary motor cortex	36	0	36	397	4.29
Insula	38	−8	6	7	3.75
Paracentral lobule	−2	− 34	58	10	3.89
Cingulate Cortex	−6	0	38	28	4.13
*V2 resting functional connectivity*					
*Migraineurs > Controls*					
Primary auditory cortex	60	−10	−2	17	4.69
Left secondary auditory cortex	− 46	−42	12	80	5.64
Right secondary auditory cortex	64	−40	4	30	4.49
Primary somatosensory cortex	42	−22	36	6	4.36
Left secondary somatosensory cortex	−40	−10	14	76	4.56
Right secondary somatosensory cortex	58	−12	18	15	4.64

Montreal Neurological Institute (MNI) coordinates, cluster sizes, and t scores for the significant differences in the infra-slow oscillation power and resting functional connectivity analyses

### Functional connectivity

#### V1 and V2 connectivity

**Fig. 2:**
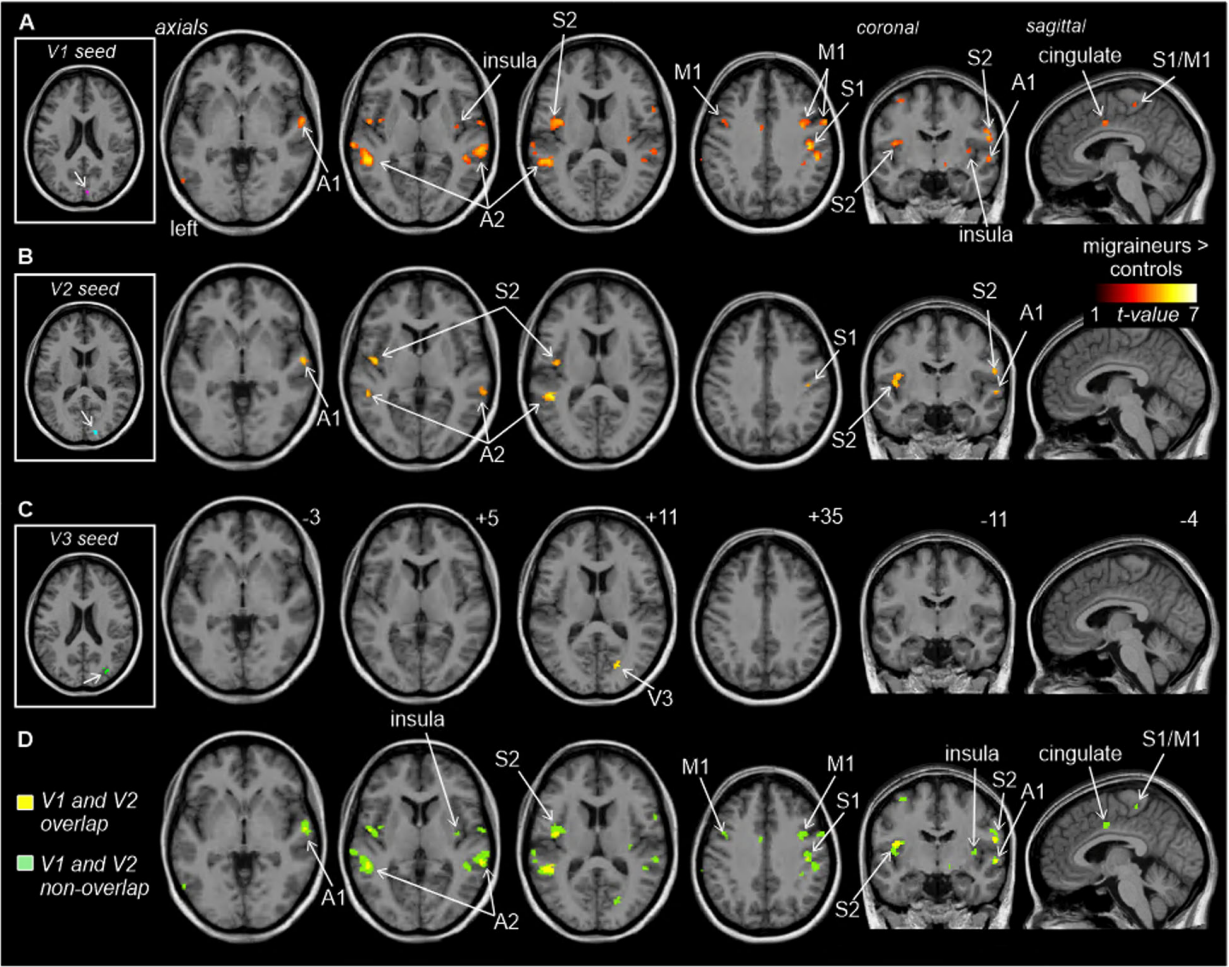
Significant differences in resting right V1, V2 and V3 connectivity between controls (*n* = 71) and migraineurs (*n* = 32) (random effects, *p* < 0.05, corrected). **A**. (left) An overlay of the right V1 seed extracted from the ALFF analysis (pink). Regions in which connectivity strength is significantly greater in migraineurs compared to controls overlaid on a mean T1-weighted anatomical image set. **B**. (left) An overlay of the right V2 seed (blue). Significant increased connectivity in migraineurs compared to controls, overlaid on a mean T1-weighted anatomical image set. **C**. (left) An overlay of the right V3 seed (green). Significant increased connectivity in migraineurs compared to controls, overlaid on a mean T1-weighted anatomical image set. **D**. Overlap (yellow) and non-overlap (green) of significant clusters in V1 and V2 functional connectivity analyses, overlaid on a mean T1-weighted anatomical image set. Location of each axial, coronal and sagittal slice in Montreal Neurological Institute (MNI) space is indicated at the top right of each slice. Slice locations are the same for all three seeds. *A1: primary auditory cortex; A2: secondary auditory cortex; M1: primary motor cortex; S1: primary somatosensory cortex; S2: secondary somatosensory cortex; V1: primary visual cortex; V2: secondary visual cortex; V3: third visual complex*

Functional connectivity analysis of the four visual cortex seeds revealed significantly greater connectivity strengths compared with controls in a number of brain regions for three of the seeds (Fig. 2A-C; Table 2). Whilst no significant differences emerged for the left V3 seed and the right V3 seed resulted in a significant difference in only a small region within the right V3 itself, the right V1 and right V2 seeds displayed significant connectivity strengths differences in a number of brain regions. Both V1 and V2 displayed significantly greater connectivity in migraineurs with the primary auditory (A1) and secondary auditory (A2) cortices and the primary somatosensory (S1) and secondary somatosensory (S2) cortices. In addition, the right V1 also displayed greater connectivity in migraineurs with the insular cortex, i.e., in the region that processes gustatory information, primary motor cortex (M1) and the cingulate cortex. In no region was connectivity strength greater in controls compared with migraineurs.

None of the four seeds displayed significantly greater resting connectivity strengths in controls compared with migraineurs.

**Fig. 3:**
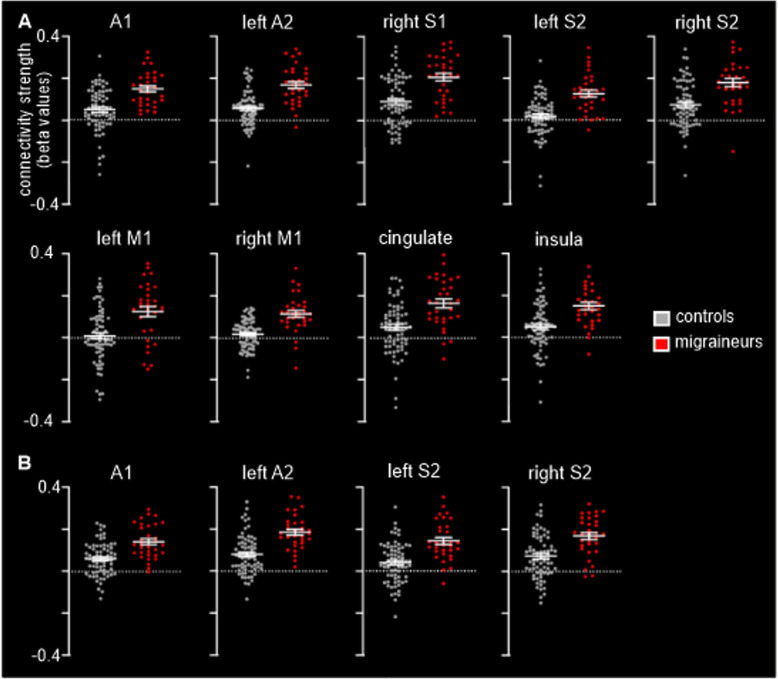
**A**. Plots of individual and mean (and standard error mean) connectivity strengths in the clusters derived from the V1 functional connectivity analysis. **B**. Plots of individual and mean (and standard error mean) connectivity strengths in the clusters derived from the V2 functional connectivity analysis. *A1: primary auditory cortex; A2: secondary auditory cortex; S1: primary somatosensory cortex; S2: secondary somatosensory cortex; M1: primary motor cortex; V1: primary visual cortex; V2: secondary visual cortex*

Although the right V1 connectivity differences were more extensive than the V2 connectivity differences, there was considerable overlap (Fig. 2D; Table 2). In migraineurs, both V1 and V2 clusters displayed significantly greater connectivity strengths with auditory processing cortices such as the right A1 (mean ± SEM connectivity strength controls vs migraineurs: *V1 seed* 0.05 ± 0.01 vs 0.15 ± 0.01; *V2 seed* 0.06 ± 0.01 vs 0.14 ± 0.01), left A2 (*V1 seed* 0.06 ± 0.01 vs 0.17 ± 0.02; *V2 seed* 0.08 ± 0.01 vs 0.19 ± 0.01) and right A2 (*V1 seed* 0.07 ± 0.01 vs 0.17 ± 0.02; *V2 seed* 0.08 ± 0.01 vs 0.18 ± 0.02) (Fig. 3). In migraineurs, both V1 and V2 seeds also displayed greater connectivity strengths with somatosensory processing cortices such as the right S1 (*V1 seed* 0.09 ± 0.01 vs 0.21 ± 0.02; *V2 seed* 0.07 ± 0.01 vs 0.17 ± 0.02), the left S2 (*V1 seed* 0.02 ± 0.01 vs 0.13 ± 0.02; *V2 seed* 0.04 ± 0.01 vs 0.14 ± 0.02) and right S2 (*V1 seed* 0.08 ± 0.01 vs 0.18 ± 0.02; *V2 seed* 0.07 ± 0.01 vs 0.17 ± 0.02) (Fig. 3).

In addition, the right V1 seed also displayed significantly greater connectivity strength in migraineurs in the cortical region that processes gustatory information, the insular cortex (0.05 ± 0.01 vs 0.15 ± 0.02), as well as the left M1 (0.01 ± 0.02 vs 0.13 ± 0.02), right M1 (0.02 ± 0.01 vs 0.12 ± 0.02), the paracentral lobule, i.e. S1/M1 (0.12 ± 0.02 vs 0.25 ± 0.03) and in a multi-modal sensory processing region, the cingulate cortex (0.05 ± 0.02 vs 0.17 ± 0.02) (Fig. 3A). Importantly, close inspection of connectivity values from all significant clusters for both the V1 and V2 seeds reveals that overall, controls displayed connectivity strengths close to zero, whereas in all areas migraineurs displayed positive connectivity strengths.

## Discussion

Our findings provide evidence for altered resting connectivity patterns between major sensory processing cortices in migraineurs. More specifically, we found in migraineurs during the interictal phase, increased infra-slow oscillatory power and furthermore, these visual regions displayed significantly increased coupling with auditory, gustatory, somatosensory, multisensory and motor cortical regions, suggesting altered global processing of sensory information. These changes likely underpin the altered visual, auditory, gustatory and somatosensory processing observed in migraineurs even between migraine events.

Whilst the neural mechanisms underlying disruptions to sensory processing during a migraine event, particularly visual processing, has long been a topic of interest, few human investigations have explored the neural mechanisms responsible for the subtle changes in sensory processing during the interictal phase when migraineurs are pain free. A recent resting-state fMRI connectivity study explored cortical networks associated with visual, auditory and somatosensory processing in interictal migraine [27]. They provided evidence for widespread disturbances in the processing of these sensory modalities, and interestingly found that primary sensory areas maintained local functional connectivity but displayed impaired connectivity with higher-order association areas [27]. This was corroborated by another resting-state fMRI connectivity study in which interictal migraineurs were found to exhibit sensorimotor network dysfunction predominately between the S1 and premotor areas [28]. Furthermore, a recent resting-state fMRI connectivity study reported that during the interictal phase, the hypothalamus modulates activity of visual processing areas [29]. We have previously revealed that during the premonitory phase, migraineurs exhibited an increase in infra-slow oscillatory power in pain-modulating regions that were found to be involved in the ictal phase of migraine, i.e. dorsal pons, hypothalamus and the midbrain periaqueductal gray (PAG) [17]. Whilst in this previous study we did not find significant differences in the interictal group at the same statistical threshold as the premonitory group, in this current study we focused our attention on visual cortices given that it is known that there are more subtle changes in visual processing during the interictal compared with premonitory phases of migraine. This restricted analysis revealed increased infra-slow oscillatory power within discrete regions of the visual cortex in migraineurs during the interictal phase. Evidence suggests that infra-slow oscillations are maintained by cyclic gliotransmitter release [30, 31] and it has been shown that astrocytes can exhibit pacemaker oscillations at frequencies between 0.03–0.06 Hz and these waves can propagate among surrounding astrocytes [30, 32, 33]. It has been hypothesized that in pathological conditions, greater numbers of astrocytes display enhanced calcium-wave synchrony and amplitude and that impaired astrocytic activity in general can alter local synaptic sensitivity [30, 31, 34–36]. It is possible that on-going glial-neural interactions alter synaptic sensitivity in visual cortices, which may underpin the diminished thresholds for light and reduced visual discomfort thresholds [8, 9], and may also predispose the system to more robust changes during a subsequent migraine event.

Interestingly, we found that the most extensive coupling between visual and other sensory cortices emerged from V1 and less so V2 and not from V3. Whilst V1 and V2 have reciprocal connections, V2 also sends feedforward connections with V3-V5, where it splits into dorsal and ventral streams, which specialize in processing different aspects of visual information [37]. Although it has been previously proposed that visual cortex hyperexcitability underpins ictal and not interictal photophobia [38], our data suggests that altered visual cortex function may also underlie interictal photophobia. Whilst V1, V2 and V3 show subtle changes in on-going activity oscillations, only V1 and V2 display altered connections with other sensory processing regions, including those that process auditory, somatosensory and gustatory information.

In addition to subtle changes in visual processing, the interictal phase is associated with subtle changes in auditory, gustatory and somatosensory processing. Interictal phonophobia is reported by more than half of migraineurs and many migraineurs report reduced auditory discomfort thresholds [8, 9]. The ability to recognize, identify and make sounds involves perceptual and cognitive processes [39] and in a study comparing central auditory processing performance, migraineurs in the interictal phase were reported to perform worse in auditory gap detection, in the discrimination of short and long duration as well as temporal processing [40]. Furthermore, phonophobia was found to be associated with cutaneous allodynia [14], highlighting changes in interconnectivity between sensory modalities in migraineurs. It is also known that somatosensory processing is altered in migraineurs during the interictal phase. For example, one study reported that interictal migraine brains are characterized by a habituation deficit of cortical evoked responses to repetitive, non-noxious sensory stimuli and that this deficit normalized during a migraine attack [7]. Further, multiple electrophysiological studies of migraine report functional changes between attacks, including hyperresponsivity to repeated sensory stimuli leading to cutaneous allodynia, abnormal recruitment of neuronal networks and impaired habituation [7]. The S1 was shown to exhibit an increase in cerebral blood flow during the interictal phase [41] and has been found to be thicker than controls, in a cohort that also showed diffusional abnormalities in the ascending trigeminal somatosensory pathway [42]. Though the precise role of the S1 in migraine pathophysiology remains unclear, it is evident that processing in the S1 is altered in individuals between migraine attacks and the significant increase in connectivity strength with other sensory processing cortical regions in interictal migraineurs suggests altered multi-sensory integration.

In addition, the perception of taste is processed in the anterior insular cortex, an area that also responds to olfactory stimulation [43], and there are reports that in migraine patients, the insula may be involved in the aversive response or disgust to food to protect from symptoms of nausea and vomiting [44]. Whilst osmophobia is less common than photophobia and phonophobia, there is evidence that migraineurs in the interictal phase experience olfactory hypersensitivity [45]. Though abnormal excitability and integration between visual, auditory, olfactory/gustatory and somatosensory cortices are relatively well-accepted, there are conflicting reports regarding abnormal excitability of motor cortices. In a transcranial magnetic stimulation study, interictal migraineurs were found to exhibit hyperexcitability in the occipital cortex but not the motor cortex [46]. The role of the motor cortex in migraine requires further study.

Although we are confident in the robustness of our data, there are several limitations to note. Firstly, we have not collected information regarding altered sensory perception in our migraineurs during the interictal phase. If we would have done so, we could have related any changes in connectivity strengths between sensory regions with changes in sensory percepts. In future investigations, we aim to collect such data so that we can relate it directly to changes in sensory cortex activity and connectivity. Secondly, we have not explored data of migraineurs in the ictal phase. Given that photophobia and phonophobia takes place during the headache in a more robust manner, it would be interesting to compare the ongoing functional activity within and between sensory cortices in interictal and ictal states. Future studies investigating the activity of the visual pathway in ictal states could certainly consolidate this data. Finally, though we did not detect any significant linear relationship between migraine frequency and duration with connectivity changes, we only included migraineurs with less than eight migraines per month. Extending the migraine cohort to a wider range of frequencies, including chronic migraineurs who suffer from over 15 headaches (with at least eight of these migraines) per month, could reveal a relationship between frequency and brain changes which could be underlying high-frequency episodic and chronic migraine. This certainly requires further investigation.

## Conclusions

These data provide evidence for a dysfunctional sensory network in pain-free migraine patients which may be underlying altered sensory processing between migraine attacks. We have provided data for the disruption of ongoing activity patterns in the visual cortex and altered connectivity within cortical regions that process various types of sensory information in pain-free interictal migraineurs. These findings are consistent with the idea that hypersensitivities to individual stimuli and migraine are not independent. Furthermore, our data supports the finding in migraine that increased sensitivity to one sensory stimulus is associated with increased sensitivity to other sensory stimuli and may contribute to more severe migraines. Understanding how integration of multisensory information is affected even in pain-free states, could help in filling gaps in our understanding of migraine pathophysiology, and critically, the initiating events leading to a migraine attack.

## Data Availability

The data presented in this study are available on request from the corresponding author. The data are not publicly available due to ethical reasons.

## Abbreviations

V1: primary visual cortex
V2: secondary visual cortex
V3: third visual complex
PAG: midbrain periaqueductal gray
SpV: spinal trigeminal nucleus
M: male
F: female
B: bilateral
L: left
R: right
I: interictal
OCP: oral contraceptive pill
SSRI: selective serotonin reuptake inhibitor
fMRI: functional magnetic resonance imaging
BOLD: blood oxygen level dependent
SPM12: Statistical Parametric Mapping version 12
DRIFTER: Dynamic Retrospective Filtering
MNI: Montreal Neurological Institute
ALFF: amplitude of low-frequency fluctuation
FWHM: full-width half maximum
A1: primary auditory cortex
A2: secondary auditory cortex
S1: primary somatosensory cortex
S2: secondary somatosensory cortex
M1: primary motor cortex
